# Development of a quantitative NS1 antigen enzyme-linked immunosorbent assay (ELISA) for Zika virus detection using a novel virus-specific mAb

**DOI:** 10.1038/s41598-024-52123-2

**Published:** 2024-01-30

**Authors:** Stefanny Viloche Morales, Gabriela Mattoso Coelho, Taíssa Ricciardi-Jorge, Gisiane Gruber Dorl, Camila Zanluca, Claudia Nunes Duarte dos Santos

**Affiliations:** 1 Laboratório de Virologia Molecular, Instituto Carlos Chagas, FIOCRUZ, Curitiba, Paraná Brazil; 2 Viral Gene Expression Group, The Pirbright Institute, Pirbright, Surrey UK

**Keywords:** Infectious-disease diagnostics, Virology, Microbiology

## Abstract

Viruses from the *Flaviviridae* family, such as Dengue virus (DENV), Yellow fever virus (YFV), and Zika virus (ZIKV) are notorious global public health problems. ZIKV emergence in Polynesia and the Americas from 2013 to 2016 raised concerns as new distinguishing features set it apart from previous outbreaks, including its association with neurological complications and heightened disease severity. Virus detection is impaired as cross-reactivity to other closely related orthoflaviviruses is common among commercially available diagnostic kits. While non-structural protein 1 (NS1) has been used as an early marker of DENV and West Nile virus (WNV) infection, little is known about NS1 expression during ZIKV infection. In the present work, we developed a NS1 capture ELISA using a novel ZIKV-specific monoclonal antibody to study NS1 expression dynamics in vitro in mosquito and human cell lines. While detectable in culture supernatants, higher concentrations of NS1 were predominantly cell-associated. To our knowledge, this is the first report of NS1 detection in human cells despite viral clearance over time. Tests with human samples need to be conducted to validate the applicability of NS1 detection for diagnosis, but overall, the tools developed in this work are promising for specific detection of acute ZIKV infection.

## Introduction

Arboviruses are transmitted by hematophagous vectors, particularly mosquitoes, in which a part of their replicative cycle occurs. These viruses are maintained in nature predominantly through a sylvatic transmission cycle between arthropod vectors and non-human vertebrate reservoirs that act as amplifying hosts^1,2^. Since human infection is mostly incidental and produces low viremia, they act as dead-end hosts by stopping virus dissemination to a new vector. Nonetheless, spillover events can lead to the establishment of urban cycles where a few arboviruses, such as Yellow Fever (YFV), Dengue (DENV), Chikungunya (CHIKV), and Zika (ZIKV), have expanded their reach, including humans as amplifying hosts^1,2^.

Although Zika virus was first isolated in 1947 from non-human primates in Zika forest in Uganda, its circulation was mostly silent, with only sporadic cases of human infection being reported in sub-Saharan Africa and Southeast Asia before the first major outbreak in Yap Island, Micronesia, in 2007. Human infection was considered a mild, self-limiting flu-like febrile illness with a classic clinical pattern (fever, rash, arthralgia, and conjunctivitis) that would only be developed in a fraction of infected people^3,4^. However, the outbreaks in French Polynesia, Easter Island, New Caledonia, and the Cook Islands in 2013–2014^5–7^, followed by the outbreak in Brazil and the Americas in 2015–2016^8–11^, shed light on new clinical presentations that set them apart from previous outbreaks. For the first time, neurological complications in fetuses and adults were causally linked to Zika virus infection^4,12,13^. ZIKV-induced congenital diseases ranging from cerebral calcifications to microcephaly, intrauterine growth restriction, fetal death, and stillbirth were reported^14–16^. On the other hand, infection has also been associated with Guillain–Barré Syndrome (GBS), possibly as a consequence of anti-ZIKV neutralizing antibody activity, causing nerve damage and leading to an inflammatory demyelinating polyneuropathy^17,18^. While the number of ZIKV cases declined considerably after 2016, transmission is still documented in some countries, with the Americas being the region with highest number of reported cases annually. According to the most recent WHO report, eighty nine nations and territories have reported evidence of autochthonous mosquito-borne ZIKV transmission as of December 2021. Despite the high morbidity due to severe sequelae resulting from infection, critical gaps remain in our understanding of Zika virus biology.

ZIKV is an enveloped positive-stranded RNA virus in the *Flaviviridae* family. The 11-kb RNA genome encodes a polyprotein that is subsequently processed by viral and host proteases to generate structural (capsid, pre/membrane, and envelope) and seven non-structural proteins (NS1, NS2A, NS2B, NS3, NS4A, NS4B, and NS5)^19^. Studies with closely related flaviviruses showed that the non-structural protein 1 (NS1) is essential for the establishment of infection, having an important role in viral replication as well as in pathogenicity and immune system evasion^20–22^. This 46–55 kDa glycoprotein presents itself as a membrane-associated homodimer as well as a secreted hexameric lipoprotein particle^23^ that can be detected in infected individuals and correlate with disease severity. The antigen presence in sera has been used as a marker for acute infections by DENV and WNV^24–26^, making it an interesting target for early diagnosis of ZIKV infections. Despite this, little is known about NS1 production during ZIKV infection. In this study, we developed a specific quantitative NS1 antigen capture ELISA to investigate the dynamics of expression and secretion of this antigen in human and mosquito cell lines and address its applicability as a diagnostic marker of acute infection.

## Results

### Production of ZIKV-specific monoclonal antibody and recombinant NS1 protein

**Figure 1:**
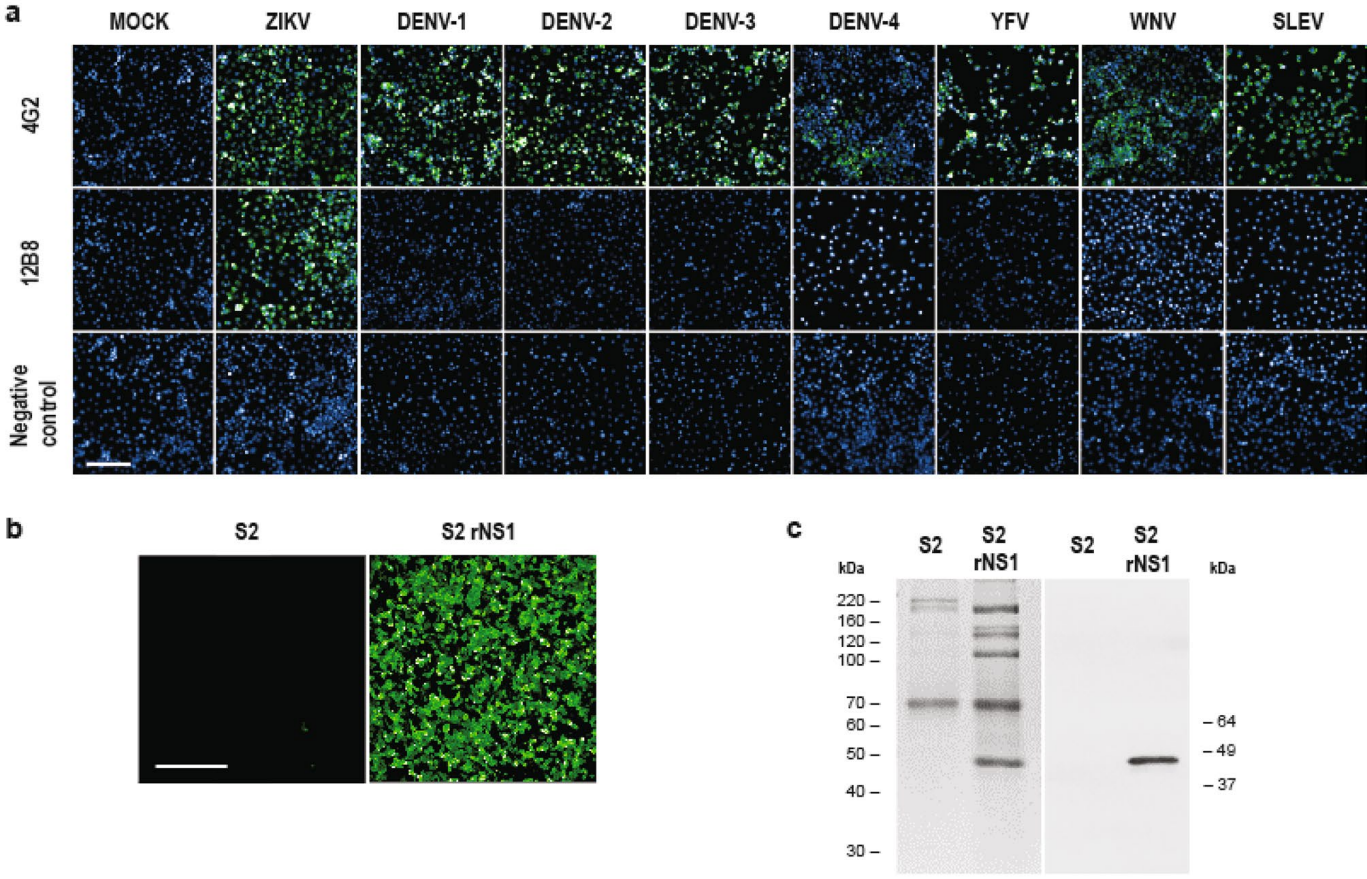
Characterization of 12B8 mAb. (**a**) IFA of C6/36 cells infected with ZIKV ZV BR 2015/15261, DENV serotypes 1–4, YFV, WNV, and SLEV. Uninfected cells (Mock) were also included as controls. In addition to mAb 12B8, cells were also stained with 4G2 and anti-GFP for positive and negative controls of infection, respectively. Reactivity was visualized with an Alexa Fluor 488-conjugated anti-mouse IgG antibody and counterstained with DAPI. Images were obtained with Operetta CLS high-content imaging system at 20 × magnification. Scale bar indicates 100 μm. (**b**) IFA of rNS1-expressing cells stained with 12B8 and Alexa Fluor 488 conjugated anti-mouse IgG antibody. Wild-type S2 cells were included as a negative control. Images were obtained with a Leica AF6000 modular system with 20 × magnification. Scale bar indicates 100 μm (**c**) Supernatant of NS1 expressing cells was purified and resolved on 10% SDS-PAGE and stained with Coomassie blue (left panel) or analyzed by western blot (right panel). The membrane was stained with 12B8 mAb and alkaline phosphatase-conjugated anti-mouse IgG. Supernatant of wild-type S2 cells were included as a negative control.

Monoclonal antibodies (mAb) were generated from the fusion of splenocytes harvested from ZIKV-immunized Balb/c mice and P3X63Ag8.653 cells. Stable hybridomas were screened for reactivity against ZIKV by immunofluorescence assay (IFA), as well as to NS1 by Western Blot (WB). Out of all positive mAbs, 12B8 was selected for its high fluorescence intensity and specificity. This mAb presented no cross-reactivity against a panel of closely related flaviviruses (DENV serotypes 1–4, YFV, WNV, and Saint Louis encephalitis virus (SLEV)) and was able to bind to both the African (see Fig. 3) and Asian lineages of ZIKV (Fig. 1a). The sensitivity of 12B8 was similar to that of pan-flavivirus mAb 4G2, used as a positive control of infection. Further characterization revealed that the 12B8 antibody was an IgG2b/κ isotype. Recombinant NS1 (rNS1) expressed in Schneider 2 (S2) insect cells was successfully detected by IFA (Fig. 1b) and WB analysis (Fig. 1c), establishing that the 12B8 mAb binds to a linear epitope.

### ZIKV NS1 antigen capture ELISA

**Figure 2:**
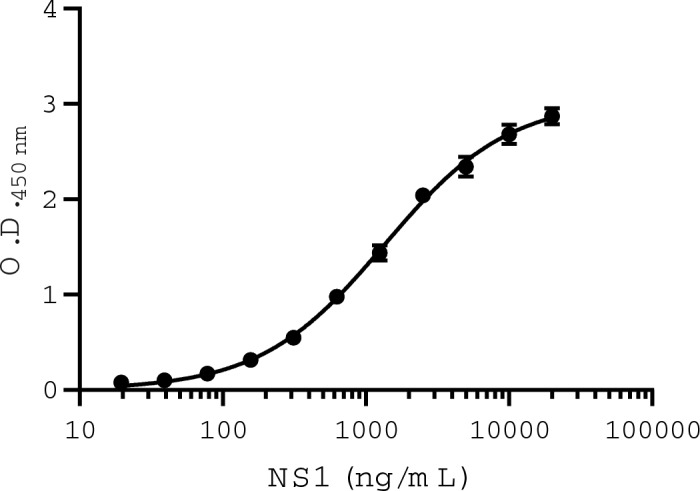
ZIKV NS1 antigen capture ELISA. Standard curve was built by serial dilutions of rNS1. Data is representative of three independent experiments and presented as mean (SD).

The test developed consisted of antigen capture and detection by 12B8 mAb associated with a standard curve of purified ZIKV rNS1 to determine protein concentration in samples. rNS1 was serially diluted from 40 μg/mL to 19.5 ng/mL to determine the assay sensitivity. The mean value of optical density at 450 nm for the blank was subtracted from the samples prior to fitting a nonlinear regression model. The limit of detection (LOD) of the test is 19.5 ng/mL, with a concentration-dependent response up to 10 μg/mL (Fig. 2). Therefore, this was the chosen range of the standard curve for interpolation of unknown values. Specificity of the test was assessed with high concentrations (10 μg/mL) of purified recombinant YFV and DENV-3 NS1 as well as with supernatant of infection (Fig. S2). No cross-reactivity was observed.

### NS1 expression and secretion in human and mosquito cell lines

**Figure 3:**
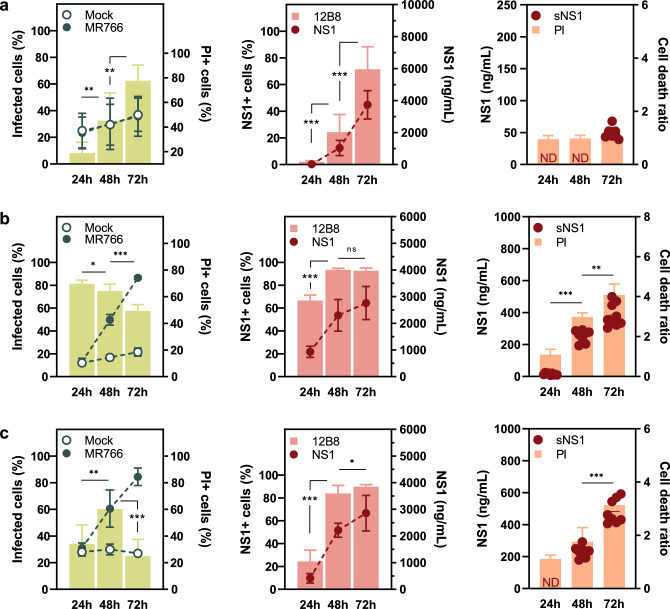
NS1 expression dynamics during ZIKV MR766 infection. (**a**) C6/36, (**b**) A549, and (**c**) JEG-3 cells were infected with a multiplicity of infection (MOI) of 1, 0.1, and 0.01, respectively. The percentage of infected and damaged cells are presented in the left panels. Green bars represent frequency of infection as determined by 4G2 staining (left axis), and lines cell viability by propidium iodide (PI) staining (mock versus infected; right axis). NS1 expression was assessed by 12B8 staining and quantitative ELISA (middle panels). The percentage of NS1 positive cells are presented as pink bars (left axis) and lines concentration of total NS1 (right axis). Soluble NS1 (sNS1) was quantified by ELISA and presented with cell death data normalized over mock (right panel). Concentration of sNS1 is presented as a scatter plot (left axis), and orange bars as cell viability (right axis). Values under LOD were considered negative (ND). Flow cytometry and ELISA data were obtained from infections carried out simultaneously. Statistical analysis of 4G2 and 12B8 positive cells (left and middle panel) and soluble NS1 (right panel) was performed with unpaired Welch’s *t*-test. Ns if p > 0.05; *if p≤ 0.05; **if p ≤ 0.01; ***if p ≤ 0.001. Data is presented as the mean (SD) of three independent experiments.

**Figure 4:**
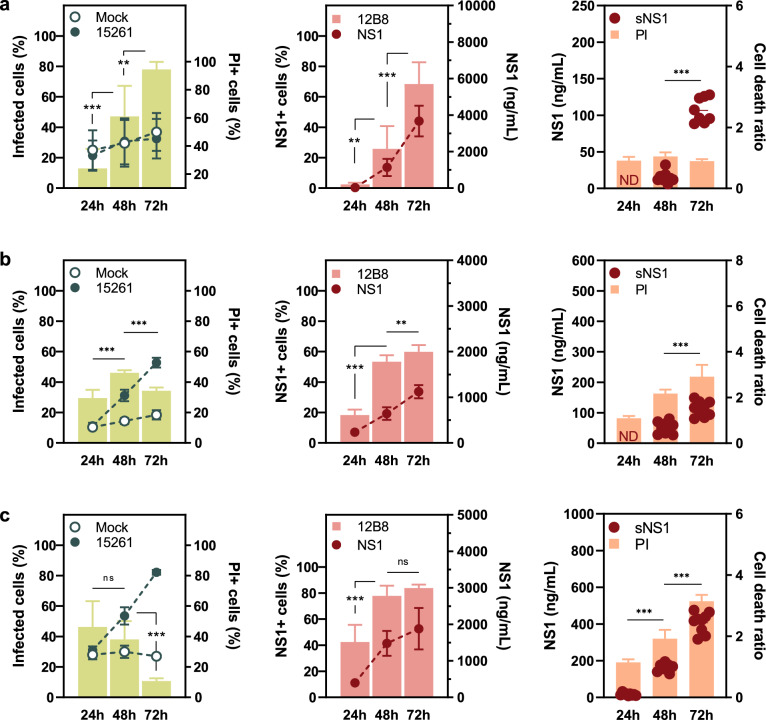
NS1 expression dynamics during ZIKV ZV BR 2015/15261 infection. (**a**) C6/36, (**b**) A549, and (**c**) JEG-3 cells were infected with a multiplicity of infection (MOI) of 1. The percentage of infected and damaged cells are presented in the left panels. Green bars represent frequency of infection as determined by 4G2 staining (left axis), and lines cell viability by propidium iodide (PI) staining (mock versus infected; right axis). NS1 expression was assessed by 12B8 staining and quantitative ELISA (middle panels). The percentage of NS1 positive cells are presented as pink bars (left axis) and lines concentration of total NS1 (right axis). Soluble NS1 (sNS1) was quantified by ELISA and presented with cell death data normalized over mock (right panel). Concentration of sNS1 is presented as a scatter plot (left axis), and orange bars as cell viability (right axis). Values under LOD were considered negative (ND). Flow cytometry and ELISA data were obtained from infections carried out simultaneously. Statistical analysis of 4G2 and 12B8 positive cells (left and middle panel) and soluble NS1 (right panel) was performed with unpaired Welch’s *t*-test. Ns if p > 0.05; **if p ≤ 0.01; ***if p ≤ 0.001. Data is presented as the mean (SD) of three independent experiments.

To study NS1 production and secretion over time, C6/36, A549, and JEG-3 cells were infected with the ZIKV strain MR766 (Fig. 3), as well as the clinical isolate from the 2015 epidemic, ZV BR 2015/15261 (Fig. 4). NS1 synthesis was assessed at 24-, 48-, and 72-h post-infection by staining infected cells with 12B8 mAb. Additionally, culture supernatant and cell lysates were analyzed with the developed ZIKV NS1-capture ELISA to determine total and soluble antigen concentration.

C6/36 cells produced the highest concentration of total NS1 among all tested cell lines. Despite this, secreted NS1 levels were low, falling outside the limit of detection of the test at the earlier timepoints (Figs. 3a and 4a, right panel). ZIKV ZV BR 2015/15261 infected cultures displayed an overall higher percentage of 4G2 positive cells when compared to MR766 infection (Figs. 3a and 4a, left panel); nonetheless, comparable levels of cell-associated NS1 and total NS1 were detected (Figs. 3a and 4a, middle panel), which could be attributed to viral load differences. Although the frequency of NS1-positive cells increased over time following a similar pattern to viral infection with both MR766 and ZV BR 2015/15261, a different trend was observed in human cell lines.

Peak infection in A549 and JEG-3 cells was detected up to 48 h in MR766 and ZV BR 2015/15261 infected cultures. At 72 h post-infection, the percentage of 4G2 positive cells declined, while NS1 positive cells maintained constant levels from 48 to 72 h (Figs. 3b,c and 4b,c, left and middle panel). This contrasting phenotype is particularly heightened in JEG-3 cells, where the frequency of NS1+ cells is 4 and eightfold higher than that of 4G2+ cells during MR766 and ZV BR 2015/15261 infections, respectively (Figs. 3c and 4c, left and middle panel).

Total and soluble NS1 quantified by ELISA increased over time in both cell types. Considering the cytopathic effect of ZIKV infection in mammalian cells, leakage of intracellular components of damaged cells could contribute to the amount of soluble NS1 detected by ELISA. Since no increase in cell death was observed during infection in C6/36 cells (Figs. 3a and 4a, right panel), it is likely that NS1 present in the supernatant originated from active secretion. On the other hand, the contribution of cell-associated antigen to total soluble NS1 cannot be ruled out, as significant cell death is observed in A549 and JEG3 cells infected with both ZIKV strains (Figs. 3b,c and 4b,c, right panel). Higher cell viability could support viral replication for a longer period and a higher viral load, thus allowing NS1 accumulation, which might be the case during C6/36 infection.

## Discussion

Flaviviruses non-structural proteins have been found to play an important role for establishment of virus infection. NS1 is a conserved glycoprotein that can be found as intracellular monomers and dimers or be actively secreted as a hexameric lipoprotein particle^23,27–32^. While it has been established that intracellular NS1 plays an essential role in negative-strand viral RNA synthesis^33–37^, secreted NS1 contributes to pathogenicity and immune system evasion. Extracellular NS1 can bind to proteins of the complement system and antagonize their functions^38–41^. Disruption of innate immune response has also been previously reported by NS1 interaction with toll-like receptors (TLR) as well as with RIG-I-like receptors (RLR) pathways^20,42–44^.

NS1 presence in sera as an early marker of infection was first described for DENV^24,25^ but was later proposed for WNV and JEV^26,45,46^, thus making an interesting target for virus specific diagnosis. Although NS1-capture ELISAs have been developed for these orthoflaviviruses, their use for diagnosis is still underutilized for viruses other than DENV. Furthermore, there is insufficient knowledge about NS1 production during ZIKV infection to effectively address the use of this protein for early virus detection.

To explore NS1 synthesis, secretion patterns, and its potential as a marker for ZIKV diagnosis, we developed a NS1-based capture ELISA for antigen quantification in mosquito and human cells cultures. The selected specific monoclonal antibody is used for capture and detection of NS1, thus favoring the binding of oligomers. Our test displayed similar sensitivity as other commercial and *in house* tests for other orthoflaviviruses^24,26,45,47–49^, with a limit of detection of 19.5 ng/mL.

Overall, we showed that NS1 appears to be predominantly associated with cells, a pattern that was also observed for YFV^50^, with notably higher levels compared to ZIKV infection. A singular study has previously assessed extracellular NS1 in ZIKV-infected Vero cells. NS1 levels were reported to be around 800 ng/mL^49^, which is significantly lower than what is observed in similar studies with WNV and YFV, where the antigen can range up to 7–9 μg/mL^45,50^. In this work, low NS1 secretion during ZIKV infection was confirmed using relevant human cell lines (A549 and JEG-3), where sNS1 was present within the 400–500 ng/mL range at later timepoints.

While for decades NS1 secretion was thought to be absent in insect cells, recent reports using more sensitive methods have detected its presence in C6/36 and Aag2 culture supernatants of DENV and YFV infected cells^50,51^. During ZIKV infection, extracellular NS1 detection was limited to later timepoints in insect cells. Despite the high percentage of infected cells at 72 h, sNS1 levels were still lower than the amount detected for human cells.

NS1 presence in sera of orthoflavivirus-infected patients is highly variable; antigen concentration can fluctuate from 0.01 to 50 μg/mL during DENV infection^25^, while for YFV, it can range from 0.1 to 4.5 μg/mL^50^. Concentration of ZIKV NS1 in human is estimated to be 30 ng/mL. Considering the low viremia observed during ZIKV infection^52^ as well as the low levels of NS1 in sera and in vitro infection, the use of this antigen as a marker for diagnosis still needs to be explored with bigger cohorts.

Although the assay settings could still be improved through tests with human samples, the tools developed in this study show potential for differential diagnostic. Aside from the developed NS1 capture ELISA, the specific monoclonal antibody generated in this study could be applied to development of a point-of-care rapid test, which could be particularly advantageous in areas with orthoflavivirus cocirculation.

The most distinguishing feature observed during in vitro ZIKV infections, was cell associated-NS1 persistence in A549 and JEG-3 while viral infection decreased over time. The drop in frequency of infected cells is associated to an increase in number of damaged cells. It is then plausible to suggest that antigen accumulation on the cell membrane could be attributed to the binding of sNS1 as well as the uptake of antigen released through cell lysis.

Previous studies have demonstrated that both secreted DENV and ZIKV NS1 can bind to the plasma membrane of uninfected cells and be endocytosed through different mechanisms^53,54^. DENV sNS1 can be detected up to 48 h within late endosomes of uninfected hepatocytes and pretreatment with this protein was also shown to increase general endocytic activity as well as DENV infection^54^. Although a recent study has presented evidence of the participation of SRB1 receptor in NS1 internalization in C6/36 and Huh7 cells^53^, NS1 binding to cells of epithelial and fibroblast origin was primarily linked to the glycosaminoglycans (GAG) heparin sulfate (HS) and chondroitin sulfate E^55^. It has been suggested that tissue-specific expression of GAG isoforms could play a role in restricting binding of NS1 to tissues associated with each orthoflavivirus particular pathogenesis.

Considering that the human cell lines used in this study are both of epithelial origin, it is possible that GAG-mediated sNS1 adhesion could play a role in this system. Additionally, JEG-3 cells infected with the clinical isolate ZV BR 2015/15261 (Asian lineage) displayed a higher percentage of NS1-positive cells compared to A549, although presenting a similar fold increase in cell death. Considering that NS1 presence in human placenta has been previously reported^56^, it is possible that this phenotype could be linked to tropism, as JEG-3 is a placenta-derived cell line.

Even though relevant research has been developed on ZIKV biology, many studies resort exclusively to the use of laboratory adapted strains, which could bias the observations of viral phenotypes, as it is believed that the accumulation of mutations might have contributed to the emergence of new clinical entities^57,58^, emphasizing the importance of working with currently circulating strains.

In conclusion, this is the first quantitative report that discriminates soluble, and cell associated NS1 expression dynamics during infection with ZIKV MR766 and clinical isolate ZV BR 2015/15261. Additional experiments would be necessary to determine if the phenotype of NS1 accumulation and persistence in cells during viral clearance is exclusive to ZIKV or can also be observed for other orthoflaviviruses. Further investigation on cell surface attachment and uptake of sNS1 needs to be conducted to explore its implication in immune response and help decipher the contribution of this multifaceted protein to pathogenesis and viral proliferation.

## Material and methods

### Cell culture and viruses

A549 (ATCC) and JEG-3 (ATCC) cells were maintained in Dulbecco’s modified Eagle’s medium/nutrient mixture F-12 Ham (DMEM F-12; Gibco) supplemented with 7% fetal bovine serum (FBS; Gibco), 100 U/mL of penicillin and 100 μg/mL of streptomycin. *Aedes albopictus* C6/36 (ATCC) cells were maintained at 28 °C in Leibovitz’s L-15 medium (Gibco) supplemented with 5% FBS, 0.26% tryptose broth (Gibco) and 25 μg/mL of gentamicin (Gibco) in the absence of CO_2_. *Drosophila melanogaster* S2 cells (Thermo Fischer Scientific) were maintained at 28 °C in Schneider’s Drosophila Medium (Gibco) supplemented with 7% FBS and 100 U/mL of penicillin and 100 μg/mL of streptomycin in the absence of CO_2_. All cell lines were routinely tested for mycoplasma contamination. Cell stocks were maintained at 37 °C, 5% CO_2_, unless stated otherwise.

Zika virus (ZIKV) viral stocks of strain MR766 and clinical isolate ZV BR 2015/15261 (isolated in 2015 from a subject in Brazil^59^) used in this study were grown and titrated in C6/36 cells. Additionally, Dengue virus 1 (DENV-1) MR, DENV-2 EN, DENV-3 290, DENV-4 LRV-13/422, Yellow Fever virus (YFV) M17/09 and 17DD, West Nile virus (WNV) E/7229/06, and Saint Louis Encephalitis Virus (SLEV) 78V6507 stocks were used. All experiments handling virus were carried out under a BSL-2 or BSL-3 containment in accordance with current biosafety guidelines.

### Monoclonal antibody production

All animal experimental procedures were conducted using protocols approved by the Fiocruz Ethical Committee on Animal Research under protocol LW-2/17, in accordance with the National Council on Animal Experimentation (CONCEA) regulations. All animal studies complied with the ARRIVE guidelines.

Young adult (4–6 weeks) Balb/c mice were kept under 12 h light/dark cycles with ad libitum feeding at the Carlos Chagas Institute Animal Facility. Mice were immunized with five doses (1–4, intraperitoneal; 5, intravenous) of 1 × 10^6^ PFU/animal of purified ZIKV ZV BR 2015/15261 particles with a 2-week interval between each dose. Splenocytes were harvested 3 days after the last dose and fused with P3X63Ag8.653 myeloma cells as previously reported^60^. Specificity of mAbs was tested by indirect immunofluorescence assay (IFA) against other orthoflaviviruses. After screening, hybridoma 12B8-2E3 was selected and cultured in 300 cm^2^ flasks. mAb were precipitated from culture supernatant with a saturated ammonium sulfate solution prior to affinity purification with HiTrap Protein G HP column (GE Healthcare). Purified mAb was conjugated with horseradish peroxidase (HRP) as previously described^61^ with minor modifications. Size exclusion chromatography of conjugated mAb was skipped and the final sample was dialyzed in PBS. Guardian Peroxidase Conjugate Stabilizer/Diluent (Thermo Fisher Scientific) was added 1:1 (v/v) to 12B8-2E3-HRP for long-term storage at 4 °C.

### Recombinant NS1 production

A consensus sequence coding for NS1 derived from a full genome ZIKV sequences alignment was optimized for expression in S2 cells and cloned into pMT/BiP/V5-His A (*Drosophila* Expression System; Invitrogen). Cells were co-transfected with the construct and selection vector pCoBlast (Invitrogen) to generate a stable culture after several rounds of blasticidin (25 μg/mL) selection. Clones were screened for V5-tag both by IFA and western blot (WB) after CuSO_4_ induction. For recombinant NS1 (rNS1) production, 1 × 10^8^ cells were seeded in 300 cm^2^ flasks and induced with 1.2 mM CuSO_4_. After 5 days, supernatant was harvested and rNS1 was purified by affinity to Ni-NTA Agarose (Qiagen). Final protein concentration was measured both by a colorimetric assay (Bio-rad Protein Assay, Bio-rad) and SDS-PAGE gel band quantification in ImageJ (National Institutes of Health, Bethesda, MD, USA).

### Kinetics of NS1 expression

C6/36, A549 and JEG3 cells were seeded at a density of 2 × 10^5^ cells per well into 24-well tissue culture plates. The next day cells were infected with a 200 μL inoculum of ZIKV MR766 or ZV BR 2015/15261 at different multiplicity of infection (MOI). After 1 h, inocula was replaced with fresh media and cells were maintained at 28 °C (C6/36) or 37 °C/5% CO_2_ (A549 and JEG-3) for 24, 48 and 72 h. NS1 was quantified from the supernatant and cell lysates of infected cultures with the NS1 antigen capture ELISA. Cells were lysed with 200 μL of cell lysis buffer (50 mM Tris pH 7.8, 150 mM NaCl, 1% NP-40 and cOmplete Protease Inhibitor cocktail 1×; Roche) and centrifuged to remove cell debris. The supernatant from this step was diluted to 600 μL with media prior to analysis by ELISA.

### Viability and intracellular staining

At each timepoint, cells were harvested and washed with PBS. After resuspending each sample in 300 μL of PBS, a third of it was transferred to a 96 well U-bottom plate and stained with Propidium Iodide 1 μg/mL (BD Biosciences) for 15 min protected from light. Samples were analyzed immediately in CytoFLEX LX (Beckman Coulter).

The remainder of each sample was fixed with 2% paraformaldehyde (PFA) for 1 h at room temperature (RT), protected from light. Cells were washed with PBS and incubated in 200 μL of Perm/Wash solution (P/W; BD Biosciences) for 30 min at 4 °C, centrifuged and incubated with 100 μL of the pan-flavivirus mAb 4G2 (1:200 v/v in P/W) or anti-ZIKV NS1 mAb 12B8-2E3 (1:400 v/v in P/W) for 1 h at 37 °C. Following two P/W washes, cells were incubated with Alexa Fluor 488-conjugated goat anti-mouse IgG (1:400 v/v in P/W; Invitrogen) for 40 min at 37 °C. Lastly, cells were washed twice with P/W and an additional time with PBS before being transferred to 96 well U-bottom plates for analysis in CytoFLEX LX. Raw data was analyzed on FlowJo v10.9 (BD Life Sciences) and graphed using GraphPad Prism 8 (Boston, MA, USA). Statistical analyses were performed with GraphPad Prism using Welch’s unpaired *t*-test assuming a confidence interval of 95%.

### NS1 antigen capture ELISA

96 well ELISA plate modules (Nalge Nunc International) were coated with 250 ng of purified 12B8-2E3 mAb per well, diluted in 0.05 M carbonate buffer (pH 9.6) overnight at 4 °C. Blocking buffer (PBS containing 0.05% Tween 20 and 10% FBS) was added to the plate for 1 h at 37 °C to minimize non-specific binding. Samples or standard curve (100 μL each) were added and incubated for 2 h at 37 °C, followed by 10 μg/mL of 12B8-2E3-HRP diluted in blocking buffer. After 1 h at 37 °C, 50 μL of the chromogenic substrate TMB (KPL) was added to each well. The reaction developed for 20 min at RT and was interrupted with the same volume of 2 M H_2_SO_4_. Unbound proteins were removed after each step by rinsing 5 times with washing buffer (PBS containing 0.05% Tween 20). Net value of optical density at 450 nm was calculated by subtracting the value obtained for the negative control. NS1 concentration was determined through interpolation from a rNS1 standard curve built from serial dilutions ranging from 10 μg/mL to 19.5 ng/mL on GraphPad Prism 8. Statistical analyses were performed with GraphPad Prism using Welch’s unpaired *t*-test assuming a confidence interval of 95%.

### Supplementary Information

Supplementary Figures.

## Data Availability

All data generated and analyzed during this study are included in this published article and its Supplementary Information files.
