# Supplementary material for: Development of a quantitative NS1 antigen enzyme-linked immunosorbent assay (ELISA) for Zika virus detection using a novel virus-specific mAb
Source: Sci Rep. 2024 Jan 30;14:2544. doi: 10.1038/s41598-024-52123-2 (PMC10827715; doi:10.1038/s41598-024-52123-2)
Supplement: Supplementary file 1 — Supplementary Figures. [file 41598_2024_52123_MOESM1_ESM.pdf]

Development of a quantitative NS1 antigen enzyme-linked immunosorbent assay (ELISA) for Zika virus detection using a novel virus-specific mAb

Stefanny Viloche Morales[1], Gabriela Mattoso Coelho[1], Taíssa Ricciardi-Jorge[2], Gisiane Gruber Dorl[1], Camila Zanluca[1] & Claudia Nunes Duarte dos Santos[1*].

[1]Laboratório de Virologia Molecular, Instituto Carlos Chagas, FIOCRUZ, Curitiba, Paraná, Brazil.
[2]Viral gene expression group, The Pirbright Institute, Pirbright, Surrey, United Kingdom.

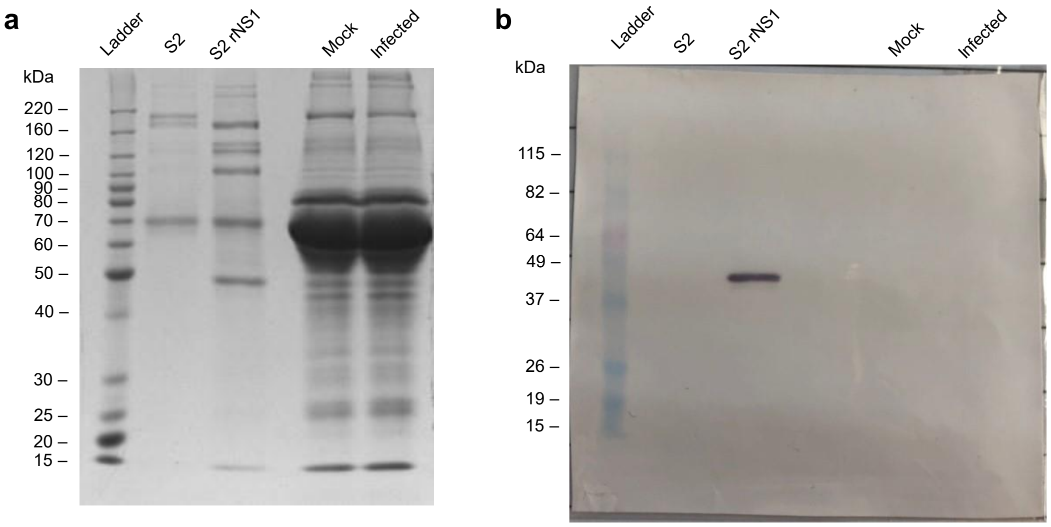

Fig. S1- Original image of SDS-PAGE and western-blot depicted in Figure 1. (a) SDS-PAGE analysis to characterize rNS1. Samples from purification of control wild-type S2 cells (S2) and S2 NS1 expressing cells were resolved in SDS-PAGE 10%. Supernatant of mock and ZIKV infected Vero cells were also tested. (b) Western blot analysis to confirm monoclonal antibody reactivity to NS1. Membrane was stained with 12B8 following with an AP conjugated secondary antibody (Promega). Reactivity was confirmed by reaction with BCIP/NBT substrate.

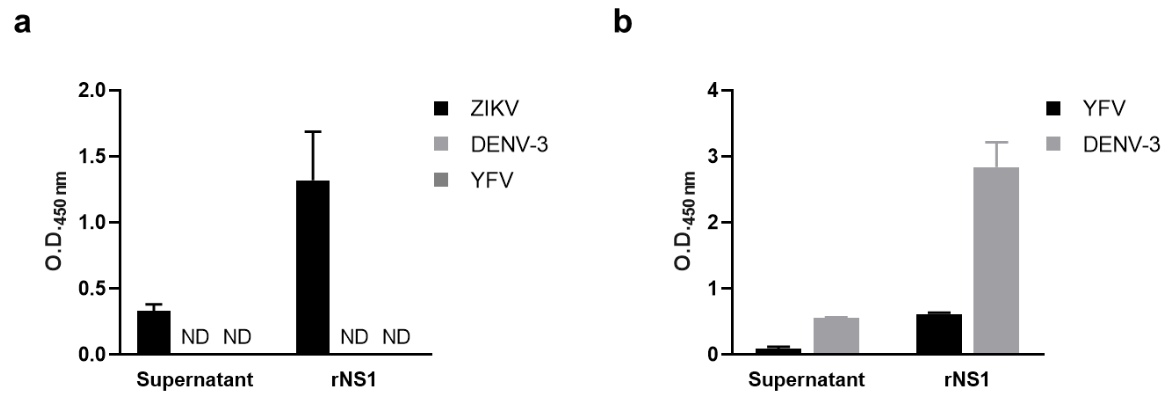

Fig. S2- The NS1-based capture ELISA does not display cross-reactivity against other orthoflaviviruses. (a) Supernatant of C6/36 cells infected with ZIKV ZV BR 2015/15261, C6/36 cells infected with DENV-3, and Vero E6 cells infected with YFV 17DD were analyzed by NS1 capture ELISA. Purified recombinant NS1 protein of ZIKV (5μg/mL), DENV-3 (10μg/mL) and YFV (10μg/mL) were used as controls. (b) The same samples were assessed by indirect ELISA, with their corresponding recombinant proteins as positive controls (10μg/mL each).
